# 474. Unlocking Knowledge: Designing an Infectious Disease Themed Escape Toom for Medical Student and Resident Education

**DOI:** 10.1093/ofid/ofaf695.162

**Published:** 2026-01-11

**Authors:** Mackenzie R Keintz, Catherine J Cichon, Evangeline Green, Jasmine R Marcelin, Benjamin Arbeiter

**Affiliations:** University of Nebraska Medical Center, Omaha, NE; Independent Scholar, Colorado Springs, Colorado; University of Nebraska Medical Center, Omaha, NE; University of Nebraska Medical Center, Omaha, NE; University of Nebraska Medical Center, Omaha, NE

## Abstract

**Background:**

Serious games like escape rooms (ER) offer immersive, interactive, and effective teaching through cooperative problem-solving and goal-oriented challenges. While ER have primarily been described in the context of medical conferences, their integration into routine medical education may serve as a novel strategy to foster early interest in infectious diseases (ID) and enhance long-term retention of medical knowledge.

**Figure 1.**
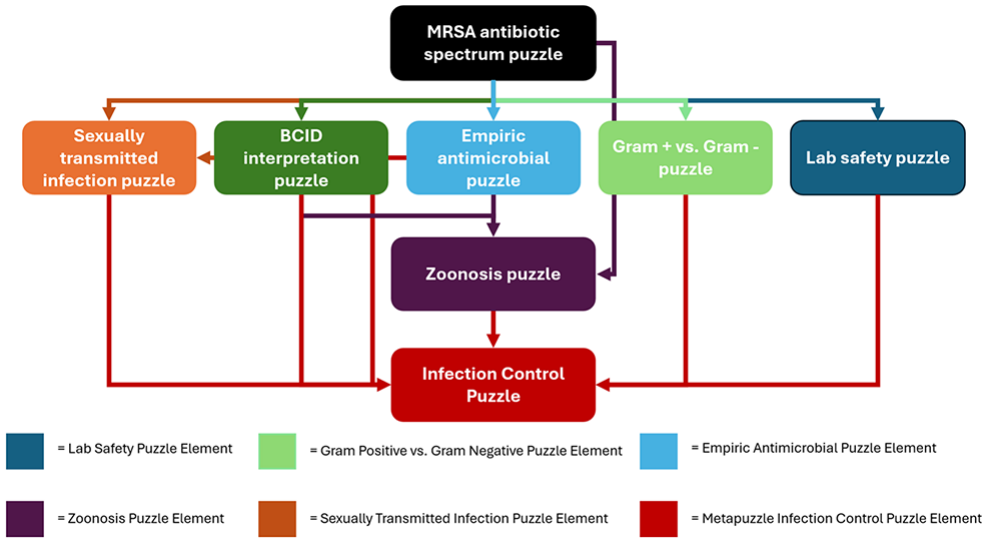
Escape room puzzle design

**Table 1.**
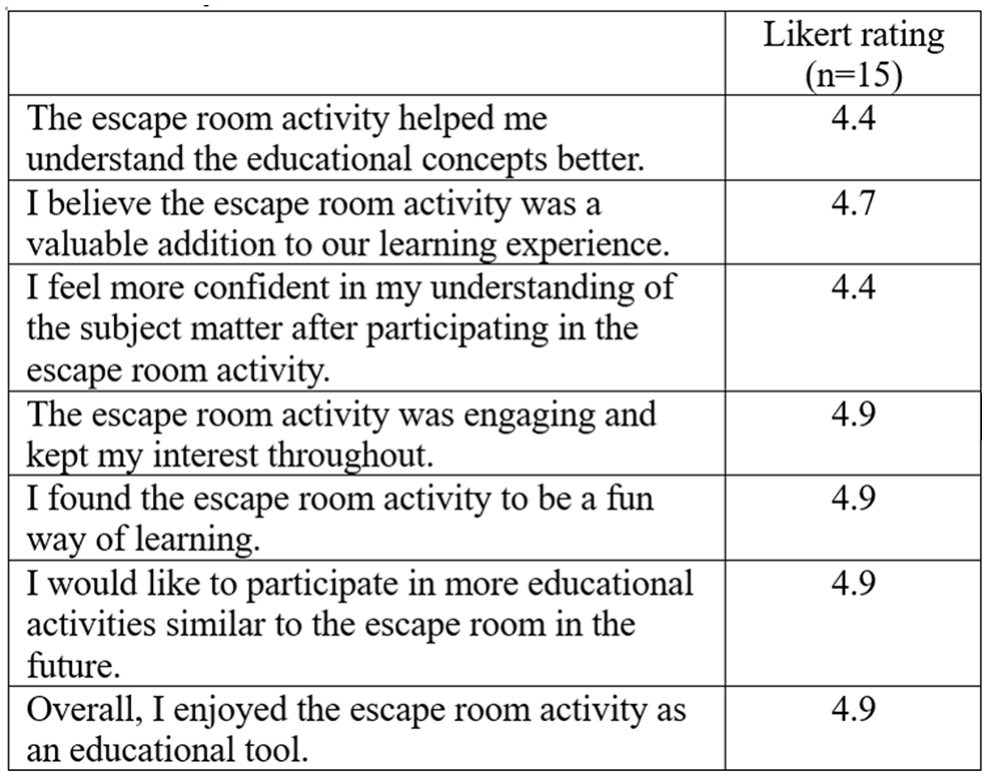
Escape room feedback via Likert-scale

**Methods:**

We designed an ID–themed ER for medical students and internal medicine residents (trainees) on the ID service, guided by Kern’s six-step approach to curriculum development. A general needs assessment was obtained via discussion with key stakeholders and literature review. A targeted needs assessment identified high yield board review exam topics and common errors in practical clinical knowledge. Eight learning objectives informed puzzle design covering key topics such as MRSA coverage, lab safety, molecular diagnostic interpretation, and empiric antibiotics (Figure 1).

We used a complex game design with sequential and open puzzle structure to maximize the number of puzzles that could be engaged simultaneously and collaboratively by 4-6 team members. The game ended when the final meta-puzzle was completed; all preceding puzzles provided clues required for the meta-puzzle to be solved. No prior ID knowledge was required; all resources were available in-room.

The 45-minute session served as the education strategy. Pre- and posttests assessments included questions across 3 categories: material introduced in ER only, lecture only, or both. A post-session Likert-scale survey measured engagement, perceived educational value and facilitating feedback for future improvements.

**Figure 2.**
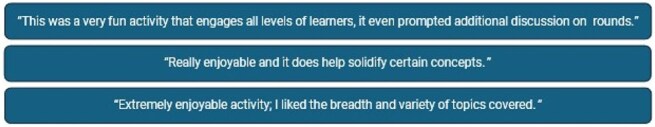
Escape room trainee feedback quotes

**Results:**

ER activity was implemented in April 2024 with 12 sessions occurring to date. Pilot data shows trainees find the activity a fun, beneficial learning experience based on survey (Table 1, Figure 2). Pre/posttest assessments ongoing.

**Conclusion:**

Escape rooms are an engaging educational tool for introducing and reinforcing ID learning objectives. The integration of these innovative educational techniques reinforces trainee engagement and fosters more positive perceptions of the ID specialty, which is an important step in building and sustaining a robust ID workforce.

**Disclosures:**

All Authors: No reported disclosures

